# Physiologically based kinetic modelling predicts the in vivo relative potency of riddelliine *N*-oxide compared to riddelliine in rat to be dose dependent

**DOI:** 10.1007/s00204-021-03179-w

**Published:** 2021-10-20

**Authors:** Frances Widjaja, Sebastiaan Wesseling, Ivonne M. C. M. Rietjens

**Affiliations:** Division of Toxicology, Wageningen University, Stippeneng 4, PO Box 8000, 6708 WE Wageningen, The Netherlands

**Keywords:** Physiologically based kinetic (PBK) model, Relative potency (REP) value, Riddelliine *N*-oxide, Riddelliine, Intestinal microbiota

## Abstract

Pyrrolizidine alkaloids (PAs) are toxic plant constituents occurring often in their *N*-oxide form. This raises the question on the relative potency (REP) values of PA-*N*-oxides compared to the corresponding parent PAs. The present study aims to quantify the in vivo REP value of riddelliine *N*-oxide compared to riddelliine using physiologically based kinetic (PBK) modelling, taking into account that the toxicity of riddelliine *N*-oxide depends on its conversion to riddelliine by intestinal microbiota and in the liver. The models predicted a lower *C*_max_ and higher *T*_max_ for the blood concentration of riddelliine upon oral administration of riddelliine *N*-oxide compared to the *C*_max_ and *T*_max_ predicted for an equimolar oral dose of riddelliine. Comparison of the area under the riddelliine concentration–time curve (AUC_RID_) obtained upon dosing either the *N*-oxide or riddelliine itself revealed a ratio of 0.67, which reflects the in vivo REP for riddelliine *N*-oxide compared to riddelliine, and appeared to closely match the REP value derived from available in vivo data. The models also predicted that the REP value will decrease with increasing dose level, because of saturation of riddelliine *N*-oxide reduction by the intestinal microbiota and of riddelliine clearance by the liver. It is concluded that PBK modeling provides a way to define in vivo REP values of PA-*N*-oxides as compared to their parent PAs, without a need for animal experiments.

**Supplementary Information:**

The online version contains supplementary material available at 10.1007/s00204-021-03179-w.

## Introduction

Pyrrolizidine alkaloids (PAs) are toxic plant secondary metabolites that may negatively impact human and animal health through accidental consumption of contaminated food. PAs are being widely distributed in almost 3% of the world’s flowering plants (Smith and Culvenor 1981), and PAs can be found in honey, teas, herbal infusions, plant supplements and contaminated salads (Deinzer et al. 1977; Fu et al. 2001; HuxTable 1980; Molyneux et al. 2011; Roulet et al. 1988; Stillman et al. 1977). More than 660 identified PA structures exist of which many occur in their *N*-oxide form (Fioeoen 2000; Fu et al. 2001; Stegelmeier et al. 1999).

In particular, PAs and PA-*N*-oxide s with 1,2-unsaturated necine bases are toxic. They can be metabolically activated by cytochromes P450 (CYP450) to form pyrrole esters known as dehydropyrrolizidine alkaloids (DHPAs), [(±)6,7-dihydro-7-hydroxy-1-hydroxymethyl-5H-pyrrolizine] (DHP), and other reactive metabolites (Chu et al. 1993; Fu et al. 2001, 2004). These metabolites react with cell proteins and DNA, forming pyrrole adducts (Chan et al. 2003; Fu et al. 2010). Pyrrole-protein and pyrrole-DNA adducts may cause hepatotoxicity (Yang et al. 2016) and genotoxicity (Fu et al. 2004; Yang et al. 2001).

Although *N*-oxidation of PAs is considered to represent a detoxification pathway, PA-*N*-oxide s are easily reduced back into the corresponding parent PAs especially by the intestinal microbiota or enzymes in liver, while reduction in intestinal tissue may be relatively less substantial (Allemang et al. 2018; Chou et al. 2003; Lindigkeit et al. 1997; Mattocks 1971; Miranda et al. 1991; Tang et al. 2013; Yang et al. 2019). Subsequently, these formed parent PAs cause the widely recognized PA type of toxicity including hepatotoxicity (Mattocks and White 1971; Mattocks 1971; Yang et al. 2017), genotoxicity and related carcinogenicity (Chou et al. 2003; Fu et al. 2001; Wang et al. 2005).

Several studies reported different findings on the relative toxicity of PA-*N*-oxide s as compared to their parent PAs. He et al. (2017) measured the amount of DNA adducts in incubations with rat liver microsomes with either riddelliine *N*-oxide or riddelliine in vitro (He et al. 2017). The ratio between the level of DNA adduct formation amounted to 0.15, being lower upon exposure to the PA-*N*-oxide. However, this in vitro study did not consider reduction of riddelliine *N-*oxide to riddelliine by the intestinal microbiota, a reaction expected to occur upon in vivo oral dosing. In contrast, as a worst case approximation, Merz and Schrenk (2016) assigned the same relative potency (REP) value to riddelliine *N*-oxide and riddelliine, assuming equal toxicity (Merz and Schrenk 2016). These interim REP values proposed by Merz and Schrenk (2016) were also proposed by Allemang et al. (2018) and by Louisse et al. (2019) who reported in vitro studies in which the potency of the *N*-oxide s appeared to be substantially lower than that of the corresponding PAs due to the absence of efficient PA-*N*-oxide reduction in the applied in vitro assays (Allemang et al. 2018; Louisse et al. 2019). Allemang et al. (2018) defined in vitro REP values based on the potency of a series of 15 PAs, including some PA-*N*-oxide s, in the micronucleus assay performed using HepaRG cells. Although the log10 Benchmark Dose (BMD) (μM) of riddelliine *N*-oxide was significantly higher than that of riddelliine, both were assigned an equal provisional REP value of 1 because Allemang et al. (2018) recognized that the in vitro model system could not be used to define REP values for the *N*-oxide s; thus, listing the interim REP values reported by Merz and Schrenk (2016) (Allemang et al. 2018; Merz and Schrenk 2016). Similarly using HepaRG cells, Louisse et al. (2019) defined REP values based on γH2AX induction, dividing the Benchmark Concentration (BMC) of parent PAs by the BMC of the corresponding PA-*N*-oxide. Although the in vitro REP values of tested PA-*N*-oxide s obtained in this way were ≤ 0.01, the interim REP values were assigned as 1 because PA-*N*-oxide s were assumed to be completely reduced to their corresponding free base PAs (Louisse et al. 2019; Merz and Schrenk 2016). However, assigning a REP value of 1 to PA-*N*-oxide s may prove to be a worst case scenario because PA-*N*-oxide s might not be completely reduced to their corresponding free base PAs (Wang et al. 2005). The use of in vitro assays to define REP values for PA-*N*-oxide s is hampered by the fact that the in vitro models generally do not contain the intestinal microbiota important for metabolic reduction of the PA-*N*-oxide s to the corresponding PAs.

Clearly, to define REP values adequate for the in vivo situation following oral exposure, one needs to consider in vivo toxicokinetics including a role for the intestinal microbiota in reduction of the PA-*N*-oxide s. To obtain adequate insight in PA-*N*-oxide toxicity, three studies actually performed in vivo experiments orally dosing rats with riddelliine or riddelliine *N*-oxide, quantifying the level of DHP-derived DNA adducts. As shown in Table 1, a dose-corrected REP value for riddelliine *N*-oxide relative to riddelliine can be derived from the level of DHP-derived DNA adducts in the liver, amounting to 0.36 and 0.41 in the two studies that quantified the adduct levels by ^32^P post- labelling (Chou et al. 2003; Wang et al. 2005) and 0.64 in another study in which the DNA adducts were quantified by LC–MS/MS (Xia et al. 2013). For the data reported by Xia et al. (2013), a dose correction was not required since these authors tested equimolar dose levels of riddelliine *N*-oxide and riddelliine. However, given the large number of PA-*N*-oxide s for which data on their relative potency are missing, alternative testing strategies to enable quantification of in vivo REP values would be of use.

**Table 1:** REP values for riddelliine *N*-oxide relative to riddelliine as derived based on literature data

Model system	Exposure	Level of DHP-dG and DHP-dA adducts/10^8^ nucleotides	Dose corrected adducts level^a^	Dose corrected REP value^b^	References
Female rats Levels of DHP-dG and DHP-dA adducts/10^8^ nucleotides in the liver detected by HPLC-ES-MS/MS	3 consecutive days 8.8 mg/kg bw (24 μmol/kg bw) riddelliine *N*-oxide	8.47	8.47	0.64	(Xia et al. 2013)
	3 consecutive days 8.4 mg/kg bw (24 μmol/kg bw) riddelliine	13.2	13.2		
Female rats Levels of DHP-DNA adducts/10^7^ nucleotides in the liver detected by ^32^P-postlabeling/HPLC	3 consecutive days 1.0 mg/kg bw (2.7 μmol/kg) riddelliine *N*-oxide	3.99	4.29	0.36	(Wang et al. 2005)
	3 consecutive days 1.0 mg/kg bw (2.9 μmol/kg bw/day) riddelliine	11.8	11.8		
Female rats Levels of DHP-DNA adducts/10^7^ nucleotides in the liver detected by ^32^P-postlabeling/HPLC	3 consecutive days 1.0 mg/kg bw (2.7 μmol/kg) riddelliine * N*-oxide	3.99	4.29	0.41	(Chou et al. 2003)
	3 consecutive days 1.0 mg/kg bw (2.9 μmol/kg) riddelliine	10.4	10.4		

^a^A dose correction was performed for the data reported by Wang et al. (2005) and Chou et al. (2003), since these studies did not apply equimolar dose levels. The dose correction was performed assuming a linear relationship between dose and level of adducts

^b^Calculated as level of adducts upon dosing riddelliine *N*-oxide divided by level of adducts upon dosing riddelliine

**Fig. 1:**
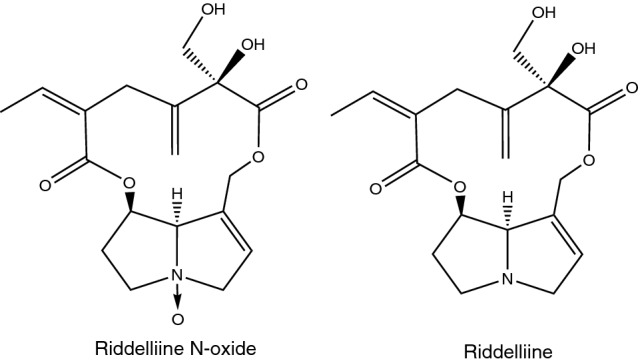
Chemical structure of riddelliine *N*-oxide and its parent PA riddelliine

The aim of the present study was to derive a REP value for a PA-*N*-oxide relative to its parent PA using in vitro-derived toxicokinetic data and in silico physiologically based kinetic (PBK) modelling. This approach would be in line with the replacement, reduction and refinement (3Rs) in animal testing. To generate a first proof of principle, riddelliine *N*-oxide was chosen as the PA-*N*-oxide and riddelliine as parent PA (Fig. 1), and the PBK model was built for rat. For this PA-*N*-oxide, in vivo data in rats already referred to above are available to evaluate the PBK model-based predictions for the REP values. To meet this goal, a previously developed PBK model for riddelliine in rat (Chen et al. 2018, 2019) was extended to include compartments for riddelliine *N*-oxide kinetics, enabling description of reduction of riddelliine *N*-oxide to riddelliine by the intestinal microbiota and in the liver, and of excretion via glomerular filtration. Subsequent comparison of the predicted area under the riddelliine concentration time curves (AUC_RID_) in blood upon oral administration of equimolar amounts of riddelliine or riddelliine *N*-oxide provided a way to predict the REP value for riddelliine *N*-oxide relative to riddelliine.

## Materials and methods

### Materials and standard chemicals

Riddelliine (98%) and riddelliine *N*-oxide (95%) were purchased from Phytolab (Phytolab GmbH & Co. KG, Germany). Dimethyl sulfoxide (DMSO) was obtained from Acros Organics (Geel, Belgium). Acetonitrile (UPLC/MS grade) and methanol were obtained from Biosolve (Valkenswaard, the Netherlands). Di-potassium hydrogen phosphate trihydrate (*K*_2_HPO_4_.3H_2_O) and potassium dihydrogen phosphate (KH_2_PO_4_) were purchased from Merck (Darmstadt, Germany). The reduced form of nicotinamide adenine dinucleotide phosphate (NADPH) was obtained from Carbosynth (Carbosynth, UK). Pooled liver and small intestine S9 from male Sprague–Dawley (SD) rats were obtained from Corning (Amsterdam, the Netherlands) and Xenotech (Kansas City, USA), respectively. Phosphate-Buffered Saline (PBS) was purchased from Gibco (Paisley, USA). Fresh fecal samples from Wistar rats (seven males and six females) were a gift from BASF (Ludwigshafen, Germany). In BASF, the samples were weighed, and transferred into anaerobic 10% v/v glycerol in PBS and were subsequently mixed and diluted to give a 20% w/v final fecal concentration. Next, these samples were filtered using sterile gauze under anaerobic conditions, and the resulting fecal slurry aliquots were stored at − 80 °C until use.

### Anaerobic rat fecal incubations

The conditions for anaerobic rat fecal incubations with riddelliine *N*-oxide were optimized with respect to linearity in time and with respect to the amount of fecal sample as previously described (Wang et al. 2020; Yang et al. 2019). Using the optimized linear conditions, the riddelliine *N*-oxide concentration-dependent rate of its reduction to riddelliine was quantified. The optimal incubation was performed for two hours in anaerobic PBS (pH 7.4), containing (final concentrations) 0.01 g feces mL^−1^, and 1–50 µM riddelliine *N*-oxide. Riddelliine *N*-oxide was added from 100 times concentrated stock solutions in DMSO (final DMSO concentration 1% (v/v)). The total incubation volume was 100 µL for both the full mixture and the controls. Solvent controls (blanks) were prepared without riddelliine *N*-oxide (replaced by DMSO), while negative controls contained no fecal slurry (replaced by PBS). Negative controls were performed for each concentration of riddelliine *N*-oxide. Samples were incubated in an anaerobic chamber (Sheldon, Cornelius, USA) at 37 °C under atmospheric conditions of 85% (v/v) N_2_, 10% (v/v) CO_2_, and 5% (v/v) H_2_. Upon 5 min preincubation at 37 °C the reactions were started by the addition of riddelliine *N*-oxide followed by 2-h incubation. After 2 h, the reaction was terminated by adding nine volumes of ice-cold methanol. Next, samples were put on ice for 5 min and were centrifuged at 21,500 *g* for 15 min at 4 °C to separate protein precipitate and supernatant. Collected supernatants were stored at − 20 °C prior to LC–MS/MS analysis for quantification of riddelliine *N*-oxide and riddelliine. All measurements were performed in triplicate.

### Aerobic incubations with rat liver and small intestine S9 fractions

Incubations of riddelliine *N*-oxide with male SD rat liver and small intestine S9 fractions were optimized as previously described (Chen et al. 2018; Wang et al. 2020; Yang et al. 2019). The optimal incubation conditions were as follows (final concentrations): 0.1 M potassium phosphate (pH 7.4), 2 mM NADPH, 0–200 µM riddelliine *N*-oxide, and with either 1 mg/mL liver S9 or 2 mg/mL small intestine S9. Riddelliine *N*-oxide was added from 100 times concentrated stock solutions in DMSO (final DMSO concentration 2%). The total incubation volume was 100 µL. Solvent controls were prepared without riddelliine *N*-oxide (replaced by DMSO), while negative controls contained no NADPH (replaced by potassium phosphate). Negative controls were performed for each substrate concentration. Upon 1 min preincubation at 37 °C, the reactions were started by the addition of riddelliine *N*-oxide. Samples were incubated for 1 h under aerobic conditions in a shaking water bath at 37 °C. The reaction was terminated by adding 20% (v/v) ice-cold acetonitrile. Subsequently, the samples were put on ice for 5 min and were centrifuged at 16,000 × *g* for 5 min at 4 °C to separate protein precipitate and supernatant. Collected supernatants were stored at − 20 °C prior to LC–MS/MS analysis for quantification of riddelliine *N*-oxide and riddelliine. All measurements were performed in triplicate.

### LC–MS/MS analysis

Riddelliine *N*-oxide and riddelliine were quantified by liquid chromatography with tandem mass spectrometry (LC–MS/MS) as previously described (Ning et al. 2019). Analysis was performed on a Shimadzu Nexera XR LC-20AD XR UHPLC System coupled with a Shimadzu LCMS-8045 mass spectrometer. 1 µL aliquot of supernatant was loaded onto a reverse phase C18 column (Phenomenex 1.7 µm 2.1 × 50 mm). The flow rate was 0.3 mL/min and the mobile phase was made with ultrapure water with 0.1% (v/v) formic acid and acetonitrile containing 0.1% (v/v) formic acid. A linear gradient was applied from 0 to 100% acetonitrile in 7 min. This percentage was kept for 1 min, after which it was reduced back to the starting conditions of 0% acetonitrile in 1 min and was equilibrated for another 4 min before the next injection. Under these conditions, riddelliine eluted at 3.8 min and riddelliine *N*-oxide at 3.9 min. For detection a Shimadzu LCMS-8045 triple quadrupole with electrospray ionization (ESI) interface was used. The instrument was operated in positive ionization mode in the multiple reaction monitoring (MRM) mode with a spray voltage of 4.0 kV. Riddelliine *N*-oxide was monitored at the [M + H] + of precursor to products of 365.95 → 94.00 (CE = − 49 eV), 365.95 → 118.05 (CE = − 36 eV) and 365.95 → 120.05 (CE = − 36 eV). Riddelliine was monitored at the [M + H] + of precursor to products of 349.95 → 94.10 (CE = − 37 eV), 349.95 → 120.10 (CE = − 30 eV) and 349.95 → 138.00 (CE = − 29 eV). Compound concentrations were quantified based on calibration curves prepared using commercially available standards.

### Determination of kinetic constants

Kinetic constants were obtained from the riddelliine *N*-oxide concentration-dependent rate for conversion of riddelliine *N*-oxide to riddelliine. The constants *V*_max_, and *K*_m_ were determined by fitting the data to the Michaelis–Menten model using GraphPad (GraphPad Prism Software version 5.04, San Diego California USA),

$$ v\, = \,V_{\max } / \, \left( {1\, + \,K_{m} /\left[ S \right]} \right)\, = \,V_{\max } *\left[ S \right]/ \, \left( {K_{m} \, + \,\left[ S \right]} \right);{{\rm  standard\, Michaelis-Menten \,equation,}} $$

where *v* is the rate of reaction, *V*_max_ is the maximum velocity expressed in µmol h^−1^ g feces^−1^, *K*_m_ is the Michaelis–Menten constant in µM, and *S* is the substrate concentration in µM.

When the concentration-dependent rate of riddelliine *N*-oxide reduction did not show saturation up to the highest concentration tested, the data were fitted to a linear equation with the slope of the linear curve representing the first-order rate constant k which equals *V*_max_/*K*_m_ when [S] <  < *K*_m_.

$$ v\, = \,k \, \left[ S \right] ; \, k\, = \,V_{\max } /K_{m} . $$

Riddelliine concentrations detected in the full incubations were corrected for the amounts detected in the corresponding blank incubations.

### Determination of fraction unbound

The fraction unbound (*F*_ub_) of both compounds was determined based on their octanol–water partition coefficient (log P) and molecular weight, using the QIVIVE Tools (www.qivivetools.wur.nl) designed by Wageningen Food Safety Research (Lobell and Sivarajah 2003; Punt et al. 2020).

### PBK model for riddelliine *N*-oxide and riddelliine in rat

**Fig. 2:**
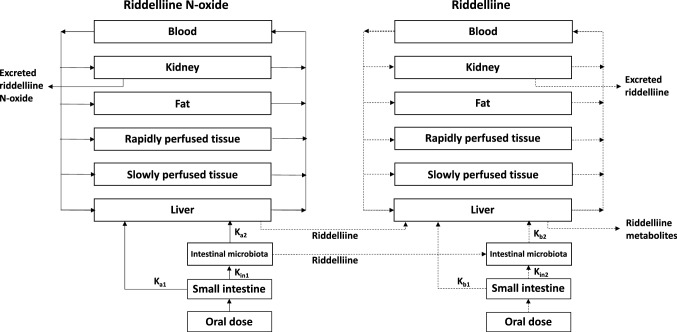
Schematic structure of the PBK model for riddelliine *N*-oxide with a submodel for riddelliine

Figure 2 shows a schematic presentation of the PBK model for riddelliine *N*-oxide in rat with a submodel for riddelliine. The PBK model included three types of parameters: (1) physiological parameters, (2) physicochemical parameters and (3) kinetic parameters. The values of these parameters are shown in Table 1S in the supplementary material. Physiological parameters, such as tissue volumes and blood flows, were obtained from the literature (Brown et al. 1997). Physicochemical parameters, such as tissue/blood partition coefficients, were calculated using the QIVIVE Tools based on a previously described method (Berezhkovskiy 2004), using log P values of -0.4 and 0.2 for riddelliine *N*-oxide and riddelliine, respectively. The values of log P (log Kow -0.4 for riddelliine *N*-oxide and log Kow 0.2 for riddelliine) were taken from PubChem as computed by XLogP3 3.0 (PubChem release 2021.05.07) because there is an absence of measured values. The method was calibrated using 8199 organic compounds, and was later tested on 406 FDA-approved small-molecule drugs and 219 oligopeptides (Cheng et al. 2007). XLogP3 was proven to produce more accurate predictions compared to its predecessors and some other methods with average unsigned errors of 0.24–0.51 units (Cheng et al. 2007). Kinetic parameters included absorption and metabolism constants, particularly kinetic constants for the reduction of riddelliine *N*-oxide to riddelliine in the liver and by the intestinal microbiota, and rate constants for uptake from the gastrointestinal tract.

The schematic structure of the PBK model for riddelliine *N*-oxide with a submodel for riddelliine was based on the previously published riddelliine PBK model with several modifications. First, the riddelliine model was connected to the model for riddelliine *N*-oxide. Riddelliine *N*-oxide enters the small intestine via oral administration and is partly absorbed in the small intestine to reach the liver via the portal vein. Second, upon passing to the large intestinal compartment riddelliine *N*-oxide is reduced to riddelliine by the intestinal microbiota. Also following uptake in the large intestine, riddelliine *N*-oxide is reduced to riddelliine in the liver compartment. Although some reduction might take place in small and large intestinal tissue, this reduction in intestinal tissue appeared to be significantly lower or even negligible compared to reduction by the intestinal microbiota and in the liver as shown in Table 3 of the result section. Consequently, reduction in intestinal tissue was not included in the model. Upon reduction of riddelliine *N*-oxide the generated riddelliine enters the riddelliine submodel. The conceptual model can also still accommodate oral absorption of riddelliine itself using only the riddelliine submodel and by adding a dose of riddelliine via an oral dose and the small intestine compartment. Third, a kidney compartment was added to both the riddelliine *N*-oxide model and the riddelliine submodel model to provide an opportunity to account for urinary excretion given that both PAs and PA-*N*-oxide s are quickly excreted in urine albeit with somewhat different efficiency (Lindigkeit et al. 1997; Powis et al. 1979; Swick et al. 1982). Urinary excretion was described by glomerular filtration (Noorlander et al. 2021) using the following equations:

$$ \begin{aligned}& {\rm GF}_{\rm RIDO} = {\rm glomerular filtration of RIDO } (\mu {\rm mol h}^{{ - {1}}} ), \\ & {{\rm GF}}_{{{{\rm RIDO}}}} ^{\prime} = {{\rm GFR}}*\left( {{{\rm CVK}}_{{{{\rm RIDO}}}} *{{\rm Fub}}_{{{{\rm RIDO}}}} } \right), \\ \,{{\rm Init GF}}_{{{{\rm RIDO}}}} = 0, \\ & {{\rm GFR}} = 0.0{8}\left( {{{\rm L h}}^{{ - {1}}} } \right), \\ & {{\rm Fub}}_{{{{\rm RIDO}}}} = 0.{994}{{.}} \\ \end{aligned} $$

$$ \begin{aligned} {{\rm GF}}_{{{{\rm RID}}}} \, & = \,{{\rm glomerular \,filtration\, of\, RID }}(\mu {{\rm  mol\, h}}^{{ - {1}}} ). \\ & {{\rm GF}}_{{{{\rm RID}}}} ^{\prime} = {{\rm GFR}}*\left( {{{\rm CVK}}_{{{{\rm RID}}}} *{{\rm Fub}}_{{{{\rm RID}}}} } \right), \\ & {{\rm Init\, GF}}_{{{{\rm RID}}}} = 0, \\ & {{\rm GFR}} = 0.0{8 }\left( {{{\rm  L h}}^{{ - {1}}} } \right), \\ & {{\rm Fub}}_{{{{\rm RID}}}} = 0.{71}0. \\ \end{aligned} $$

In these equations, G*F*_RIDO_ and G*F*_RID_ present glomerular filtration of riddelliine *N*-oxide or riddelliine (µmol h^−1^), GFR presents the glomerular filtration rate (0.0052*BW*60 L h^−1^) (Walton et al. 2004), and Fub_RIDO_ and Fub_RID_ present the fraction unbound of riddelliine *N*-oxide and riddelliine calculated as described above to amount to 0.994 and 0.710 respectively. The lower *F*_ub_ of riddelliine reflects the higher lipophilicity and resulting higher level of protein binding of riddelliine than of its *N*-oxide.

The model started with either riddelliine *N*-oxide or riddelliine entering the small intestine after an oral exposure. For model evaluation by comparison to available literature data, the dose of riddelliine *N*-oxide was set to 20 mg/kg bw riddelliine *N*-oxide as this was the dose used by Yang et al. (2019) (Yang et al. 2019), and the dose of riddelliine was set to 10 mg/kg bw of riddelliine as used by Williams et al. (2002) (Williams et al. 2002). For determination of the REP value, equimolar oral dose levels of 8.8 mg kg^−1^ bw riddelliine *N*-oxide and 8.4 mg kg^−1^ bw riddelliine (Xia et al. 2013) were considered as these doses represent the dose levels used in the in vivo rat studies available for evaluation of the predictions of the REP value.

The rate constant for transfer of both riddelliine *N*-oxide and riddelliine from the small intestine into the intestinal microbiota compartment *K*_in_ was 0.46 h^−1^ as reported by Kimura and Higaki (2002) and as used by Wang et al. (2020) (Kimura and Higaki 2002). The rate constant for absorption of riddelliine from the intestinal microbiota compartment to the liver *K*_b_ was 0.72 h^−1^ as reported by Chen et al. (2018). The rate constant for absorption of riddelliine *N*-oxide from the intestinal microbiota compartment to the liver *K*_a_ was 0.23 h^−1^, which was calculated from a previously published apparent permeability coefficient (P_app_) value based on a comparison to riddelliine (Chen et al. 2018; Yang et al. 2020) using the following equation:

$$ P_{{{\rm app RIDO}}} /k_{{{\rm a RIDO}}} \, = \,P_{{{\rm app RID}}} /k_{{{\rm a RID}}} . $$

Reduction of riddelliine *N*-oxide was modelled in two compartments of the riddelliine *N*-oxide model: reduction by intestinal microbiota in the large intestine lumen, and reduction in the liver. The riddelliine formed in the intestinal microbiota compartment was transferred to the intestinal microbiota compartment of the riddelliine submodel. The riddelliine formed in the liver compartment was transferred to the liver compartment of the riddelliine submodel. The model equations were coded and numerically integrated in Berkeley Madonna 9 (UC Berkeley, CA, USA) using Rosenbrock’s algorithms for stiff systems to predict AUC_RID_. When lacking experimental values, the blood plasma ratio (B/P) is often assumed to be 1 for basic compounds or 0.55 (1- hematocrit) for acidic compounds (Cubitt et al. 2009). Both riddelliine *N*-oxide and riddelliine are considered as basic compounds, hence the B/P ratio is assumed to be 1.

### Sensitivity analysis

A sensitivity analysis was performed to assess which parameters of the PBK model have the largest impact on the predicted area under the riddelliine blood concentration time curve (AUC_RID_). Normalized sensitivity coefficients (SCs) were calculated using the following equation:

$$\mathrm{SC}= \frac{({C}^{^{\prime}}-C)}{({P}^{^{\prime}}-P)} x \frac{P}{C}.$$

In this equation, *C* is the initial value of the model output, *C*’ is the modified value of the model output with a 5% increase of an input parameter, *P* is the initial parameter value and *P*’ is the parameter value with an increase of 5%. For the sensitivity analysis, only one parameter was changed each time, while the other parameters were kept at their initial values. A large SC value indicates that this parameter has a large impact on the predicted AUC_RID_. A dose of 8.8 mg/kg bw riddelliine *N*-oxide or 8.4 mg/kg bw riddelliine was used for the sensitivity analysis, with either 15% or 100% bioavailability for both conditions.

## Results

### Optimization of anaerobic fecal incubations

**Fig. 3:**
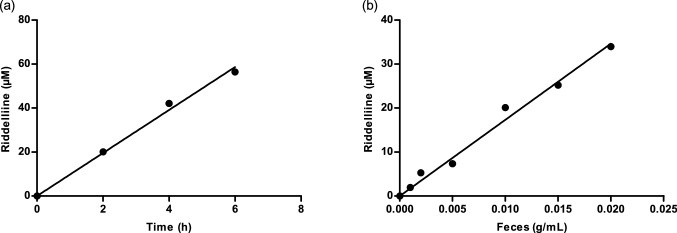
Conversion of riddelliine *N*-oxide to riddelliine in anaerobic fecal incubations with 100 µM riddelliine *N*-oxide with **a** increasing incubation time and 0.01 g/mL feces and **b** increasing amount of feces (g/mL) for 2 h

Figure 3 shows the time and fecal amount dependent conversion of riddelliine *N*-oxide to riddelliine in anaerobic incubations with rat fecal samples. From these results, it follows that for all time points and fecal concentrations tested, riddelliine formation was linear with the amount of fecal sample and with time up to at least 6 h. Based on these results, optimum conditions for subsequent kinetic experiments were selected as 0.01 g fecal sample/mL and 2 h of incubation, at which maximum conversion would not exceed 20%.

### Determination of kinetic parameters for fecal microbial metabolism

**Fig. 4:**
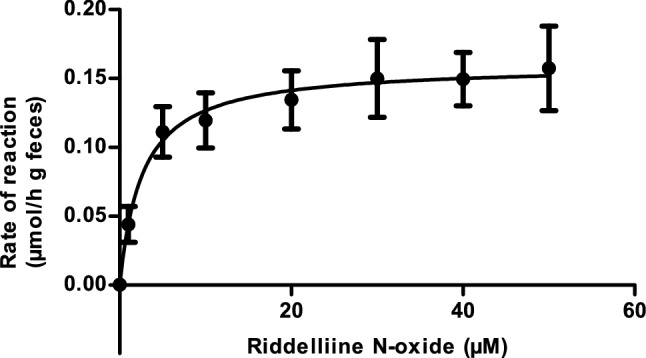
Riddelliine *N*-oxide concentration-dependent rate of riddelliine formation in anaerobic fecal incubations. Data are presented as mean ± SD of three independent experiments (n = 3)

Figure 4 presents the riddelliine *N*-oxide-dependent rate of conversion of riddelliine *N*-oxide to riddelliine in anaerobic fecal incubations under the optimized conditions. The graph reveals saturation behavior following Michaelis–Menten kinetics. Fitting the data to the Michaelis–Menten equation provided the kinetic constants *V*_max_ expressed in µmol h^−1^ g feces^−1^ and *K*_m_ in µM as well as the catalytic efficiency (*V*_max_/*K*_m_). Using the scaling factor of 0.0164 as fraction of feces to bodyweight expressed in g feces g bw^−1^ (Hoskins and Zamcheck 1968; Wang et al. 2020) and a body weight of 250 g, the *V*_max_ was converted to an in vivo *V*_max_ expressed in µmol h^−1^ per rat, which was the value used in the PBK model. The in vivo *K*_m_ was considered to be similar to the *K*_m_ obtained in vitro. Table 2 presents the obtained values.

**Table 2:** Kinetic parameters of riddelliine formation by intestinal microbiota

Parameter	Intestinal microbiota
*V*_max_ (µmol h^−1^ g^−1^ feces)	0.160 ± 0.004
Scaled *V*_max_ (µmol h^−1^)^a^	0.656 ± 0.016
*K*_m_ (µM)	2.63 ± 0.36
Catalytic efficiency (mL h^−1^ g^−1^ feces)^b^	60.84
Scaled catalytic efficiency (L h^−1^)	0.249

^a^Scaled *V*_max_ = *V*_max_*fbw*bw*1000, where fbw is fraction of feces to bodyweight (0.0164 g feces g bw^−1^) and bw is body weight of rat (0.25 kg) and 1000 to convert bw in kg to bw in gram

^b^Catalytic efficiency was calculated as *V*_max_/*K*_m_

### Determination of kinetic parameters for aerobic S9 metabolism

**Fig. 5:**
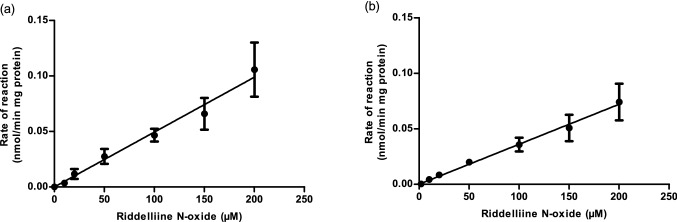
Riddelliine *N*-oxide concentration-dependent rate of riddelliine formation in aerobic incubations with S9 of **a** rat liver and **b** rat small intestine. Data are presented as mean ± SD of four independent experiments (n = 4)

Figure 5 shows the riddelliine *N*-oxide concentration-dependent rate of conversion of riddelliine *N*-oxide to riddelliine in aerobic incubations with both rat liver and small intestine S9. The obtained results reveal that in these incubations up to riddelliine *N*-oxide concentrations of 200 µM the reduction of riddelliine *N*-oxide to riddelliine does not saturate and increases linear with the substrate concentration. Thus, kinetics for riddelliine *N*-oxide reduction by liver and intestinal samples can be described by first order kinetics with a first order rate constant being equal to the slope of the curve representing *V*_max_/*K*_m_. The first-order rate constants obtained in vitro, expressed in mL min^−1^ mg S9^−1^ were scaled to the in vivo situation using scaling factors of 143 mg S9 g liver tissue^−1^ and 38.6 mg S9 g intestine tissue^−1^ (Cubitt et al. 2011; Medinsky et al. 1994; Wang et al. 2020), and liver and small intestine tissue weights of 8.5 and 3.5 g. Table 3 presents the obtained values. Given that reduction of PA-*N*-oxide s may be favored by low oxygen conditions, anaerobic kinetic parameters of riddelliine *N*-oxide reduction following Michaelis–Menten kinetics were determined as well from the results shown in Fig. 1S in the supplementary material and Table 3.

**Table 3:** Kinetic parameters of riddelliine *N*-oxide reduction in incubations with S9 of rat liver (aerobic and anaerobic) and small intestine, and the related scaled in vivo parameters

Parameter	Liver (aerobic)	Liver (anaerobic)	Small intestine
*V*_max_ (nmol min^−1^ (mg S9)^−1^)	n.a	0.296 ± 0.034	n.a
Scaled *V*_max_ (µmol h^−1^)^a^	n.a	21.58 ± 2.49	n.a
*K*_m_ (µM)	n.a	155.5 ± 33.4	n.a
Catalytic efficiency (mL min^−1^ (mg S9)^−1^)^b^	0.0005	0.0019	0.0004
Scaled catalytic efficiency (L h^−1^)	0.037^c^	0.139^c^	0.003^d^

^a^Scaled *V*_max_ = (*V*_max_/1000)*60*S9L*L*bw, where S9L is the scaling factor for the liver S9 fraction (143 mg S9 protein/g liver), L is fraction weight of rat liver (34 g/kg bw) and bw is body weight of rat (0.25 kg), 60 is to convert minutes to hours and 1000 to convert nmol to µmol

^b^Catalytic efficiency was calculated as *V*_max_/*K*_m_

^c^Scaled catalytic efficiency = (*V*_max_/*K*_m_ /1000)*60*S9L*L*bw, where S9L is the scaling factor for the liver S9 fraction (143 mg S9 protein/g liver), L is fraction weight of rat liver (34 g/kg bw) and bw is body weight of rat (0.25 kg), 60 is to convert minutes to hours and 1000 to convert mL to L

^d^Scaled catalytic efficiency = (*V*_max_/*K*_m_ /1000)*60*S9I*I*BW, where S9I is small intestinal S9 fraction (38.6 mg S9 protein/g small intestine), I is fraction weight of rat small intestine (14 g/kg bw) and bw is body weight of rat (0.25 kg) 60 is to convert minutes to hours and 1000 to convert mL to L

Tables 2 and 3 show the scaled in vivo catalytic efficiencies expressed in L h^−1^, thus obtained, revealing that the efficiency for riddelliine *N*-oxide reduction decreases in the order intestinal microbiota (0.249 L h^−1^) > liver (0.037 L h^−1^) > small intestine (0.003 L h^−1^). The scaled catalytic efficiency of riddelliine *N*-oxide reduction by intestinal microbiota was 6.7-fold and 83-fold higher than that of liver and that of small intestine, respectively. Based on these findings, riddelliine *N*-oxide reduction by the small intestinal tissue was considered non-substantial and was, therefore, not included in the PBK model. Although the catalytic efficiency for riddelliine *N*-oxide reduction was 3.8-fold fold higher under anaerobic than aerobic conditions as shown in Table 3, even under anaerobic conditions, the scaled catalytic efficiency by intestinal microbiota was higher by 1.8-fold than that of liver (0.139 L h^−1^).

### Evaluation of PBK model-based predictions

**Fig. 6:**
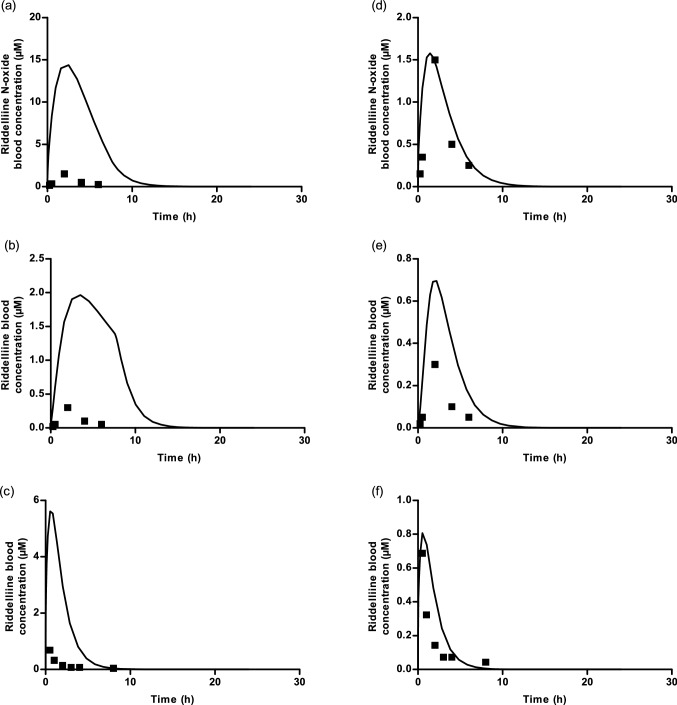
**a**, **b** Comparison of the riddelliine *N*-oxide model predictions assuming 100% bioavailability after an oral dose of 20 mg/kg bw riddelliine *N*-oxide for **a** time-dependent riddelliine *N*-oxide blood concentrations and **b** time-dependent riddelliine blood concentration with in vivo kinetic plasma concentration from Yang et al. (2019) (Yang et al. 2019). **c** Comparison of the riddelliine model predictions assuming 100% bioavailability after oral dose of 10 mg/kg bw riddelliine for time-dependent riddelliine blood concentrations with in vivo kinetic plasma concentration from Williams et al. (2002) (Williams et al. 2002). **d**, **e** Comparison of the same data as in Figure a-c assuming 15% bioavailability. The riddelliine submodel was adapted from Chen et al. (2018) (Chen et al. 2018). Continuous lines represent model prediction and black squares represent in vivo data

In vivo kinetic data were available for riddelliine *N*-oxide and riddelliine, enabling evaluation of the developed PBK model for rat by comparing the blood concentration–time curves, maximum blood concentration (C_max_) and time to reach maximum blood concentration (*T*_max_) values as predicted by the PBK model with in vivo kinetic data from literature. Yang et al. (2019) reported riddelliine *N*-oxide and riddelliine blood concentrations in rats that were orally exposed to 20 mg/kg bw riddelliine *N*-oxide (Yang et al. 2019). Figure 6a shows that the difference between the predicted and observed C_max_ and *T*_max_ of riddelliine *N*-oxide at a dose of 20 mg/kg bw was 9.6- and 1.3-fold, respectively. Figure 6b shows that the difference between the predicted and observed C_max_ and *T*_max_ of riddelliine at a dose of 20 mg/kg bw, were 6.6- and 1.8- fold, respectively. Williams et al. (2002) reported riddelliine blood concentrations in rats that were orally exposed to 10 mg/kg bw riddelliine (Williams et al. 2002). Figure 6c shows that the difference between the predicted and observed C_max_ and *T*_max_ of riddelliine at a dose of 10 mg/kg bw, were 8.2- and 1.1- fold, respectively.

To better fit the predicted data and especially the C_max_ with in vivo kinetic values, a lower bioavailability value was considered. Figure 6d shows this optimized fit which was obtained assuming 15% bioavailability resulting in a difference between the predicted and observed C_max_ and *T*_max_ of riddelliine *N*-oxide at a dose of 20 mg/kg bw, of 1.1- and 0.7-fold, respectively. Figure 6e shows that the difference between the predicted and observed C_max_ and *T*_max_ of riddelliine at a dose of 20 mg/kg bw, were 2.3- and 1.1-fold, respectively. Figure 6f shows that the difference between the predicted and observed C_max_ and *T*_max_ of riddelliine at a dose of 10 mg/kg bw, were 1.2- and 1.1-fold, respectively. Thus, with the assumed lower bioavailability the PBK model-based predictions were even more in line with the reported experimental data.

### Predicted excretion of riddelliine *N*-oxide and riddelliine using the PBK model

**Fig. 7:**
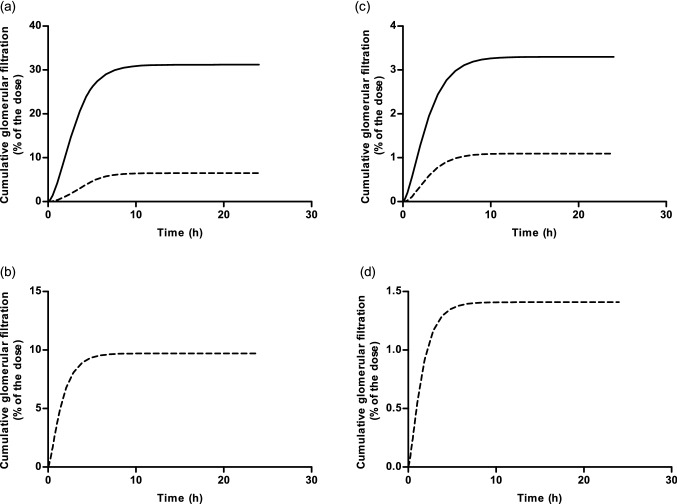
Predicted cumulative glomerular filtration as percentage of the dose (%) assuming **a**, **b** 100% bioavailability and **c**, **d** 15% bioavailability, upon dosing **a**–**c** 8.8 mg/kg bw riddelliine *N*-oxide or **b**–**d** 8.4 mg/kg bw riddelliine. Continuous lines represent glomerular filtration of riddelliine *N*-oxide, while dashed lines represent glomerular filtration of riddelliine

Upon oral administration of riddelliine *N*-oxide in the PBK model, the PBK model predicted for riddelliine *N*-oxide a different extent of glomerular filtration than for riddelliine. Figure 7a, b show the predicted cumulative urinary excretion as percentage of the dose assuming 100% bioavailability, while Fig. 7c, d present the same data assuming 15% bioavailability. Upon an oral dose of riddelliine *N*-oxide with 100% bioavailability, the cumulative urinary excretion of riddelliine *N*-oxide is 4.8-fold higher than that of riddelliine. When riddelliine is administered orally, urinary excretion of riddelliine is observed at levels that are 1.5-fold higher than the cumulative riddelliine excretion upon dosing an equimolar dose of riddelliine *N*-oxide. Upon an oral dose of riddelliine *N*-oxide and assuming 15% bioavailability, the cumulative urinary excretion of riddelliine *N*-oxide is 3.0-fold higher than that of riddelliine. When riddelliine is administered orally, urinary excretion of riddelliine is observed at levels that are 1.3-fold higher than the cumulative riddelliine excretion upon dosing an equimolar dose of riddelliine *N*-oxide.

### Derivation of a REP value for riddelliine *N*-oxide compared to riddelliine

**Fig. 8:**
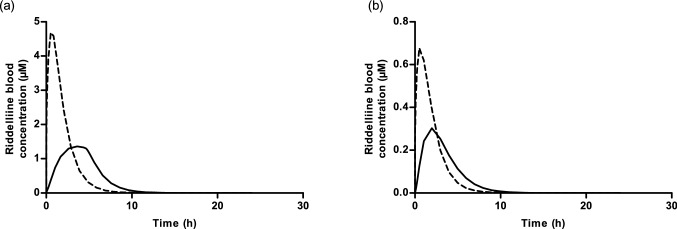
Predicted toxicokinetic profile of formed riddelliine in blood with **a** 100% and **b** 15% bioavailability. Continuous line represents predictions after an oral dose of 8.8 mg/kg bw riddelliine *N*-oxide, while the dashed lines represents prediction after oral dosage of 8.4 mg/kg bw riddelliine

Figure 8a, b show the PBK model-predicted concentration–time curves of riddelliine from rats orally dosed with 8.8 mg/kg bw riddelliine *N*-oxide or an equimolar dose of 8.4 mg/kg bw riddelliine assuming either 100% or 15% bioavailability. When bioavailability is 100%, the model predicts that upon an oral dose of riddelliine *N*-oxide the time-dependent riddelliine blood concentration shows a 3.4- fold lower C_max_ and a 6.9-fold higher *T*_max_ compared to what is observed upon an equimolar dose of riddelliine. The AUC_RID_ for rats orally dosed with riddelliine *N*-oxide is predicted to be 7.41 µM h, and for those orally dosed with an equimolar dose of riddelliine the AUC_RID_ is predicted to be 11.04 µM h. The ratio of these two AUC_RID_ values amounts to 0.67, reflecting the time-dependent difference in overall internal exposure to riddelliine, responsible for the subsequent adverse effects, and thus reflecting the potential difference in potency and, thus, the REP value of riddelliine *N*-oxide relative to riddelliine.

When bioavailability is assumed to be 15%, the AUC_RID_ for rats orally dosed with riddelliine *N*-oxide is predicted to be 1.25 µM h, and for those orally dosed with riddelliine the AUC_RID_ is predicted to be 1.60 µM h. The ratio of these two AUC_RID_ values is 0.78. These results corroborate the lower relative potency of riddelliine *N*-oxide as compared to riddelliine, while at the same time elucidating that the REP value may be dose dependent.

### An increase in dose leads to a decrease in the REP value for riddelliine *N*-oxide as compared to riddelliine

**Fig. 9:**
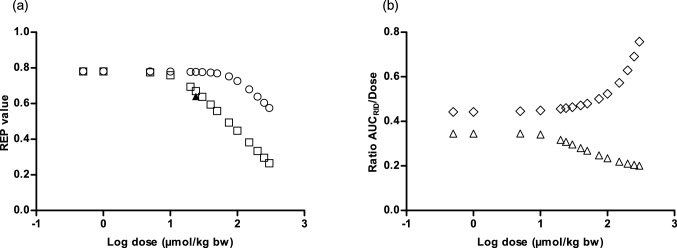
**a** REP value plotted against log equimolar dose (µmol/kg bw) of riddelliine *N*-oxide and riddelliine. White circles represents REP value assuming 15% bioavailability, white squares represent REP value assuming 100% bioavailability, and the black triangle represents the in vivo REP value extracted from Xia et al. (2013) (Xia et al. 2013). **b** Ratio of AUC_RID_/Dose against log equimolar dose (µmol/kg bw) of riddelliine *N*-oxide and riddelliine assuming 100% bioavailability. White diamonds represent the ratio AUC_RID_/dose upon dosing riddelliine, while white triangles represent the ratio AUC_RID_/dose upon dosing riddelliine *N*-oxide

Figure 9a shows further evaluation of this observation of a dose dependent REP value for riddelliine *N*-oxide as compared to riddelliine. For comparison the figure also presents the in vivo REP value reported by Xia et al. (2013) based on a study in which rats were dosed with riddelliine *N*-oxide or riddelliine detecting the levels of DHP-derived DNA adducts in the liver by LC–MS/MS. The results obtained reveal that the REP value decreases with increasing dose level, and also that the predictions adequately match the in vivo REP value extracted from Xia et al. (2013), when assuming 100% bioavailability.

To investigate the effect of assuming anaerobic liver conditions that would facilitate riddelliine *N*-oxide reduction, the dose-dependent REP value of riddelliine *N*-oxide as compared to riddelliine was also calculated using the kinetic parameters obtained in anaerobic liver incubations. The results thus obtained are presented in Fig. 2S in the supplementary material. When assuming 100% bioavailability, the anaerobic AUC_RID_ for rats orally dosed with riddelliine *N*-oxide is predicted to be 9.09 µM h, and that for rats orally dosed with an equimolar dose of riddelliine the AUC_RID_ is predicted to be 11.04 µM h. The ratio of these two AUC_RID_ provides a REP value of 0.82, which is higher than the REP value of 0.67 obtained using the kinetics in aerobic liver incubations and also higher than the in vivo REP value of 0.64 derived from the data by Xia et al. (2013).

To elucidate the reason underlying this dose-dependent effect on the REP value, Fig. 9b presents the dose-dependent AUC_RID_/dose ratio’s upon dosing either riddelliine *N*-oxide or an equimolar amount of riddelliine, the ratio of which defines the REP value. In this curve, the AUC_RID_ values are divided by the respective dose to reflect if the AUC_RID_ changes linear with the dose, resulting in a constant AUC_RID_/dose ratio with increasing dose level, or not. From these results, it follows that the REP value decreases with the dose because of two reasons; (1) with increasing dose of riddelliine *N*-oxide its reduction to riddelliine by the intestinal microbiota saturates so that the AUC_RID_ will no longer increase linear with the dose leading to decreasing AUC_RID_/dose ratio’s with increasing dose of riddelliine *N*-oxide, and (2) with increasing dose of riddelliine its clearance in the liver saturates so that the AUC_RID_ with increasing dose of riddelliine is no longer linear with the dose, leading to increasing AUC_RID_/dose ratio’s with increasing dose of riddelliine. Thus as the dose increases, the AUC_RID_ from orally administered riddelliine *N*-oxide shows a decreasing trend, while orally administered riddelliine generates AUC_RID_ with an increasing trend. Combining these two effects results in decreasing REP values with increasing dose.

### Sensitivity analysis

**Fig. 10:**
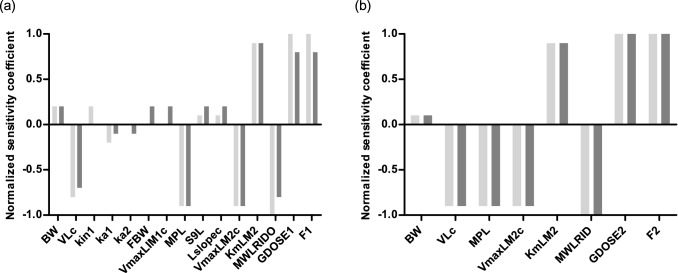
Normalized sensitivity coefficients for the parameters of the rat PBK model for **a** riddelliine *N*-oxide and **b** riddelliine on predicted AUC_RID_ in blood concentration at a single oral dose of 10 mg/kg bw. *BW* body weight of rat, *VLc* liver fraction, *kin1* transfer rate of riddelliine *N*-oxide from lower ileum to cecum, *ka1* absorption rate of riddelliine *N*-oxide from small intestine to liver, *ka2* absorption rate of riddelliine *N*-oxide from intestinal microbiota compartment to liver, *FBW* fraction of feces to bodyweight, *V*_*max*_*LIM1c* is the maximum rate for reduction of riddelliine *N*-oxide by intestinal microbiota, *MPL* liver microsomal protein yield, *S9L* liver S9 protein yield, *Lslopec* kinetic parameter for reduction of riddelliine *N*-oxide by liver (*V*_max_/*K*_m_), *VmaxLM2c and KmLM2* the maximum rate of depletion and the Michaelis–Menten constant for depletion of riddelliine in liver, *MWLRIDO and MWLRID* molecular weight of riddelliine *N*-oxide and of riddelliine, *GDOSE1 and GDOSE2* oral dose of riddelliine *N*-oxide and of riddelliine, *F1 and F2* bioavailability of riddelliine *N*-oxide and of riddelliine. Light grey bars represent sensitivity coefficients at 15%, while dark grey bars represent sensitivity coefficients at 100%

To further evaluate the performance of the developed model, a sensitivity analysis was performed to assess the parameters that affect the prediction of the AUC_RID_ in blood. The sensitivity analysis was performed at an oral dose of 8.8 mg/kg bw riddelliine *N*-oxide or 8.4 mg/kg bw riddelliine. Only the parameters that result in a normalized sensitivity coefficient higher than 0.1 in absolute value are shown in Fig. 10. For the riddelliine *N*-oxide model, the linear sensitivity analysis showed similar sensitive parameters at 15% and 100% bioavailability, except for kin1 which was more influential when assuming 15% bioavailability, and ka2, FBW and VmaxLIM1c which were more influential when assuming 100% bioavailability. Parameters that were influential under both conditions were VLc, MPL, VmaxLM2c, KmLM2, MWLRIDO, GDOSE1 and F1, reflecting parameters mainly related to clearance of formed riddelliine by the liver compartment. For the riddelliine submodel, the linear sensitivity analysis also showed similar sensitive parameters at 15% and 100% bioavailability. The sensitive parameters are in agreement with the previous sensitivity analysis performed by Chen et al. (2018) for the riddelliine model (Chen et al. 2018) except for PLL, PSL and Kb which were not found to be as influential in the current submodel.

## Discussion

The aim of the present study was to predict an in vivo REP value for riddelliine *N*-oxide relative to its parent PA riddelliine using in vitro kinetic parameters which were translated to the in vivo situation by PBK modelling. The results obtained show that the REP values for PA-*N*-oxide s relative to their parent PAs of 0.1 and 1.0 as could be derived from previous studies (Allemang et al. 2018; Bull et al. 1958; Downing and Peterson 1968; He et al. 2017; Louisse et al. 2019; Mattocks 1971; Merz and Schrenk 2016), respectively under- and overestimate the toxicity of the *N*-oxide s. A REP value of 0.1 would be obtained when using data from in vitro studies (Allemang et al. 2018; He et al. 2017; Louisse et al. 2019), but also from in vivo studies where PA-*N*-oxide s were not administered orally (Bull et al. 1958; Downing and Peterson 1968; Mattocks 1971), while a REP value of 1.0 would represent a worst case scenario approximation, assuming that the PA-*N*-oxide s would be completely reduced to the corresponding free base PAs (Merz and Schrenk 2016).

This result appears to be in line with especially the REP value derived from the in vivo study comparing the levels of DHP-DNA adduct formation in the liver by HPLC-ES-MS/MS amounting to 0.64 (Xia et al. 2013) as shown in Table 1 and Fig. 9a. Given the fact that the in vivo available REP value of 0.64 was best predicted when using the kinetic data obtained in aerobic liver S9 incubations and also the fact that the liver is unlikely to be anaerobic but known to contain on average about 3 mM oxygen (Boobis and Powis 1974), it was concluded that kinetic parameters from aerobic liver S9 incubations are preferred to predict REP values of pyrrolizidine *N*-oxide s relative to their parent PAs. Compared to the values obtained by quantifying the DHP-adduct levels by ^32^P-postlabelling upon equimolar dose correction of 0.36 (Wang et al. 2005) and 0.41 (Chou et al. 2003) the predictions are at the high end, although it should be taken into account that quantification of DNA adduct levels by ^32^P-post-labelling may be less accurate than by LC–MS/MS. Using LC–MS/MS, known to be likely more accurate than ^32^P-labelling, the ratio of DNA adducts between rats orally fed with equimolar doses of riddelliine *N*-oxide and riddelliine defining a REP value of 0.64 was closer to the PBK model-based predictions of 0.67 (100% bioavailability (Fig. 6a)) and 0.78 [15% bioavailability (Fig. 6d)].

The difference between these in vivo experimental data is unlikely due to the dose levels used in these studies which amounted to 1.0 mg/kg bw/day for both compounds (Chou et al. 2003; Wang et al. 2005) or 8.8 mg/kg bw and 8.4 mg/kg bw riddelliine (Xia et al. 2013). At an oral dose of 1.0 mg/kg bw of either riddelliine *N*-oxide or riddelliine, representing the dose levels used by Wang et al. (2005) and Chou et al. (2003) (Chou et al. 2003; Wang et al. 2005), the predicted REP value amounted to 0.74 at both 15% and 100% bioavailability, and was, thus, different from the values of 0.36 or 0.41 derived from the DHP-adduct levels determined by ^32^P-post-labelling (Table 1). At an oral dose of 8.8 mg/kg riddelliine *N*-oxide or 8.4 mg/kg bw riddelliine, the dose levels used in the vivo study reported by Xia et al. (2013) (Xia et al. 2013), the predicted REP values amounted to 0.78 at 15% bioavailability and 0.67 at 100% bioavailability, values that are both close to the value of 0.64 derived from the DHP-dG and DHP-dA adduct level determined by HPLC-ES-MS/MS (Table 1). Given the results of the present study it could be argued that at the lower dose levels (1.0 mg/kg bw), as applied in the studies of Wang et al. (2005) and Chou et al. (2003), the REP values would be expected to be higher and not lower than what would be obtained at 8.8 mg/kg riddelliine *N*-oxide or 8.4 mg/kg bw riddelliine. Thus, these calculations support that the deviation between the two studies might not be related to the difference in dose levels, but rather be related to the different analytical approaches used to quantify the DHP adducts.

A previous in vitro study quantified DHP-DNA adducts in incubations with rat liver microsomes in the presence of calf thymus DNA and equal concentrations of riddelliine *N*-oxide or riddelliine resulting in a ratio of 0.15 (He et al. 2017). The relatively low REP value coming from this in vitro study can be ascribed to the absence of reduction of the PA-*N*-oxide by the intestinal microbiota which is not part of this in vitro model system. The results of the present study clearly revealed that compared to liver and small intestine, intestinal microbiota showed the highest scaled catalytic efficiency for the reduction of riddelliine *N*-oxide to riddelliine; hence, justifying the use of REP values based on studies that take this reduction into account.

It is of special interest to note that the PBK model-based prediction for the REP value of riddelliine *N*-oxide relative to riddelliine appeared to be dose dependent with the REP value decreasing with increasing dose levels above 10 µmol/ kg bw (equal to 3.65 mg riddelliine *N*-oxide /kg bw). To the best of our knowledge this finding has so far not been reported for PA REP values, but is important given that in vivo experiments are generally performed at higher dose levels than actual human dietary exposure. This human exposure may amount to 7.5–10.1 ng/kg bw for infants, toddlers and young children, and 5.2–5.3 ng/kg bw for adults, elderly and very elderly (EFSA et al. 2017), representing maximum mean dietary exposure to total PAs as a result of use of tea and herbs for infusion. The PBK model-based approach for estimating in vivo REP values for the PA-*N*-oxide s may actually provide a unique way to study such low dose levels. The predicted REP value for riddelliine *N*-oxide at these low dose levels was 0.78.

The observation of the dose dependency of the REP value adds complexity to the definition of REP values for PA-*N*-oxide s. The results of the present study also reveal that when considering definition of REP values for PA-*N*-oxide s the role of their reduction by the intestinal microbiota and in human liver cannot be ignored, so that use of in vitro assays that do not take these toxicokinetics into account may not provide REP values representing the in vivo situation. The importance of taking differences kinetics into account when defining relative potencies of PAs was previously shown to also hold for the PAs themselves for which substantial differences in clearance may exist (Lester et al. 2019). Successful incorporation of intestinal microbiota in PBK models has been demonstrated so far in only a few other studies (Mendez-Catala et al. 2020; Wang et al. 2020) and the current study provides another proof-of-principle for this approach.

Evaluation of the current PBK model for riddelliine *N*-oxide with a submodel for riddelliine appeared to fit the available in vivo kinetic data for riddelliine *N*-oxide and riddelliine blood time profiles (Williams et al. 2002; Yang et al. 2019) reasonably well when assuming 100% bioavailability, providing a better fit assuming 15% bioavailability. The predicted REP value, however, matched the in vivo available data best when assuming 100% bioavailability (Xia et al. 2013). It is important to note that the in vivo kinetic data as reported may include some experimental inaccuracy, providing another explanation for part of the discrepancies. Yang et al. (2020) measured intestinal permeability of riddelliine *N*-oxide and riddelliine using a Caco-2 monolayer model (Yang et al. 2020). The reported Papp values for riddelliine *N*-oxide and riddelliine were 0.94 × 10^–6^ and 3.00 × 10^–6^ cm/s, respectively, which yielded a ratio of 0.31, the permeability being higher for riddelliine than for its *N*-oxide. This difference in intestinal permeability was included in the current model when defining the values for the rate of absorption of riddelliine and its *N*-oxide which were aligned with the respective Papp values. It is important to note that this difference in absorption rate between riddelliine *N*-oxide and riddelliine is not the factor responsible for the lower bioavailability of 15% of riddelliine and its *N*-oxide, which is a value that should be defined based on comparison of the AUC upon oral dosing versus the AUC upon intravenous dosing. Independent of the % bioavailability and the fit to the experimental kinetic data, the PBK model adequately predicted that the riddelliine concentration time profile upon dosing riddelliine *N*-oxide shows a lower C_max_ and higher *T*_max_ for the riddelliine blood concentration with also a relative lower AUC_RID_ than what was observed upon dosing an equimolar amount of riddelliine.

The PBK model-based approach presented allows definition of an in vivo REP value for a PA-*N*-oxide relative to its parent PA. Riddelliine is often taken as the reference PA for which the REP value is defined at 1.00 (Allemang et al. 2018; EFSA et al. 2017; HMPC 2016; Louisse et al. 2019; Merz and Schrenk 2016; Wiesner 2021). Given that both REP values of other PAs and the REP value of riddelliine *N*-oxide established in the present study are determined relative to riddelliine, the REP value of riddelliine *N*-oxide would be in line with the system for defining REP values for other PAs. In contrast to riddelliine *N*-oxide, the REP value of other PA-*N*-oxide s would first need to be determined relative to their corresponding parent PAs and later be multiplied by the REP value of these parent PAs relative to riddelliine to obtain a REP value suitable for combined exposure and risk assessment based on riddelliine equivalents.

After establishing the proof-of-principle for using this in silico–in vitro PBK modeling based approach for defining a REP value for riddelliine *N*-oxide, future research should evaluate if the established new approach methodologies (NAMs) can also adequately predict in vivo REP values for other PA-*N*-oxide s focusing in first instance on PA-*N*-oxide s for which toxicokinetic and/or in vivo toxicity data are available to enable further evaluation of the predictions made and validation of the approach. Furthermore, similar studies could be performed developing human PBK models enabling studying relative differences in potency and definition of REP values for the human situation, also providing insight in potential species differences. These human models could subsequently also be applied to define interindividual differences in the predicted relative potency of PA-*N*-oxide s as compared to the corresponding PAs. Clearly these topics are beyond the aim of the present study but provide topics of interest for future work.

In conclusion, the present study shows a novel PBK model-based approach to define in vivo REP values for PA-*N*-oxide s relative to their parent PAs using in vitro kinetic data. The model incorporates reduction by intestinal microbiota. The in vivo REP value of riddelliine *N*-oxide was predicted to be dependent on the dose levels applied and to amount to 0.78 at realistic human dietary intake levels. Altogether it is concluded that the PBK model-based approach provides a suitable novel alternative testing strategy to define in vivo REP values for PA-N-oxides.

## Supplementary Information

Below is the link to the electronic supplementary material.

Supplementary file1 (DOCX 38 kb)

## Abbreviations

3Rs: Replacement, reduction and refinement
AUC_RID_: Area under the riddelliine concentration–time curve
BMC: Benchmark concentration
BMD: Benchmark dose
*C*_max_: Maximum blood concentration
CYP450: Cytochrome P450
DHP: (±)6,7-Dihydro-7-hydroxy-1-hydroxymethyl-5H-pyrrolizine
DHPAs: Dehydropyrrolizidine alkaloids
*F*_ub_: Fraction unbound
PAs: Pyrrolizidine alkaloids
PA-*N*-oxides: Pyrrolizidine alkaloid *N*-oxides
PBK model: Physiologically based kinetic model
NAMs: New approach methodologies
REP: Relative potency
*T*_max_: Time to reach maximum blood concentration
