# Supplementary material for: Physiologically based kinetic modelling predicts the in vivo relative potency of riddelliine N-oxide compared to riddelliine in rat to be dose dependent
Source: Arch Toxicol. 2021 Oct 20;96(1):135–51. doi: 10.1007/s00204-021-03179-w (PMC8748370; doi:10.1007/s00204-021-03179-w)
Supplement: Supplementary file 1 — Supplementary file1 (DOCX 38 kb) [file 204_2021_3179_MOESM1_ESM.docx]

**Physiologically based kinetic modelling predicts the in vivo relative potency of riddelliine N-oxide compared to riddelliine in rat to be dose-dependent**

*Frances Widjaja , Sebastiaan Wesseling, Ivonne MCM Rietjens*

**Division of Toxicology, Wageningen University, PO Box 8000, 6700 EA Wageningen, The Netherlands**

*Corresponding author:

Frances Widjaja

Division of Toxicology, Wageningen University

Stippeneng 4, 6708 WE Wageningen, the Netherlands

Tel: +31-6-3451-0686

Email: [frances1.widjaja@wur.nl](mailto:frances1.widjaja@wur.nl)

**Supplementary Material**

**Table 1S**. List of parameters for PBK model of riddelliine N-oxide and of riddelliine

| **Parameter** | **Description** | **Unit** | **Value** |
| --- | --- | --- | --- |
| Physiological parameters | | | |
| BW | Body weight of rat | kg | 0.25 |
| VFc | Fraction volume of fat | - | 0.070 |
| VLc | Fraction volume of liver | - | 0.034 |
| VBc | Fraction volume of blood | - | 0.074 |
| VKc | Fraction volume of kidney | - | 0.007 |
| VRc | Fraction volume of rapidly perfused tissue | - | 0.041 |
| VSc | Fraction volume of slowly perfused tissue | - | 0.774 |
| QC | Cardiac output 15* (BW^0.74^) | L h^-1^ | 5.38 |
| QFc | Fraction blood flow to fat | - | 0.070 |
| QLc | Fraction blood flow to liver | - | 0.250 |
| QKc | Fraction blood flow to kidney | - | 0.141 |
| QRc | Fraction blood flow to rapidly perfused tissue (0.76-QLc-QKc) | - | 0.369 |
| QSc | Fraction blood flow to slowly perfused tissue (0.24-QFc) | - | 0.170 |
| Physicochemical parameters | | | |
| LogP_RIDO_ | Log P RIDO | - | -0.4^a^ |
| PF_RIDO_ | Fat/blood partition coefficient RIDO | - | 0.14^b^ |
| PL_RIDO_ | Liver/blood partition coefficient RIDO | - | 0.76^b^ |
| PK_RIDO_ | Kidney/blood partition coefficient RIDO | - | 0.83^b^ |
| PR_RIDO_ | Rapidly perfused tissues/blood partition coefficient RIDO | - | 0.84^b^ |
| PS_RIDO_ | Slowly perfused tissues/blood partition coefficient RIDO | - | 0.66^b^ |
| LogP_RID_ | Log P RID | - | 0.2^a^ |
| PF_RID_ | Fat/blood partition coefficient RID | - | 0.15^b^ |
| PL_RID_ | Liver/blood partition coefficient RID | - | 0.67^b^ |
| PK_RID_ | Kidney/blood partition coefficient RID | - | 0.72^b^ |
| PR_RID_ | Rapidly perfused tissues/ blood partition coefficient RID | - | 0.74^b^ |
| PS_RID_ | Slowly perfused tissues/ blood partition coefficient of RID | - | 0.58^b^ |
| Kinetic parameters | | | |
| kin1 | Transfer rate of RIDO from lower ileum to cecum | h^-1^ | 0.46^c^ |
| kin2 | Transfer rate of RID from lower ileum to cecum | h^-1^ | 0.46^c^ |
| ka1 | Transfer rate of RIDO from small intestine to liver | h^-1^ | 0.23^d^ |
| ka2 | Transfer rate of RIDO from intestinal microbiota compartment to liver | h^-1^ | 0.23^d^ |
| kb1 | Transfer rate of RID from small intestine to liver | h^-1^ | 0.72^e^ |
| kb2 | Transfer rate of RID from intestinal microbiota compartment to liver | h^-1^ | 0.72^e^ |
| FBW | Fraction of feces to bodyweight | g feces (g bw)^-1^ | 0.0164^f^ |
| VmaxLIM1c | Unscaled anaerobic RIDO reduction to RID by intestinal microbiota, Vmax | µmol h^-1^ (g feces)^-1^ | 0.16 |
| KmLIM1 | Anaerobic RIDO reduction to RID by intestinal microbiota, Km | µmol L^-1^ | 2.63 |
| MPL | Liver microsome protein yield | mg microsome (g liver)^-1^ | 35^g^ |
| S9L | Liver S9 fraction | mg S9 (g liver)^-1^ | 143^h^ |
| Lslopec | Unscaled aerobic RIDO reduction to RID by liver S9, Vmax/Km | mL min^-1^ (mg S9)^-1^ | 0.0005 |
| VmaxLM2c | Unscaled aerobic RID depletion by liver microsome, Vmax | nmol min^-1^ (mg microsome)^-1^ | 2.093^i^ |
| KmLM2 | Aerobic RID depletion by liver microsome, Km | µmol L^-1^ | 75.69^i^ |
| GFR | Glomerular filtration rate 0.0052*BW*60 | L h^-1^ | 0.08^j^ |
| Fub_RIDO_ | Fraction unbound of RIDO | - | 0.994^k^ |
| Fub_RID_ | Fraction unbound of RID | - | 0.71^k^ |
| Run settings | | | |
| MWL_RIDO_ | Molecular weight RIDO | g mol^-1^ | 365.4 |
| MWL_RID_ | Molecular weight RID | g mol^-1^ | 349.38 |
| GDOSE1 | Oral dose RIDO | mg (kg bw)^-1^ | 8.8^l^ |
| GDOSE2 | Oral dose RID | mg (kg bw)^-1^ | 8.4^l^ |
| F1 | Assumed bioavailability of RIDO | - | 0.15 or 1 |
| F2 | Assumed bioavailability of RID | - | 0.15 or 1 |
| Starttime | T-0 or starting time | h | 0 |
| Stoptime | T-final or ending time | h | 24 |

^a^Log Kow or Log P values were extracted from XlogP3 3.0 (Pubchem) in the absence of measured value. Log P were used to calculate partition coefficients and fraction unbound values.

^b^Partition coefficients were calculated using QIVIVE tools developed by Wageningen Food Safety Research (Punt, 2020; Berezhkovskiy, 2004).

^c^Transfer rate for both RIDO and RID from lower ileum to cecum were assumed the same as that of orally administered drugs (Kimura, 2002).

^d^Transfer rate of RIDO from small intestine and intestinal microbiota (large intestine) were assumed the same. This value was calculated based on the reported Papp value (Yang, 2020) and Ka of riddelliine (Chen, 2018).

^e^Transfer rate of RID from small intestine and intestinal microbiota (large intestine) were assumed the same. The value was extracted from previous literature (Chen, 2018).

^f^Fraction of feces to bodyweight was extracted from previous literature (Hoskins & Zamcheck, 1968).

^g^Liver microsome protein yield was extracted from previous literature (Atio, 1976)

^h^Liver S9 fraction was extracted from previous literature (Punt, 2008).

^i^RID depletion kinetic parameters were extracted from previous literature (Chen, 2018)

^j^Glomerular filtration rate for rat was calculated as derived from previous literature (Walton, 2003)

^k^Fraction unbound values were calculated using QIVIVE tools developed by Wageningen Food Safety Research (Punt, 2020; Lobell and Sivarajah, 2003)

^l^Equimolar dose of 8.8 mg/kg bw riddelliine N-oxide or 8.4 mg/kg bw riddelliine were used as those in in vivo study (Xia, 2013)

;Date : May 2021

;Purpose : PBK model riddelliine N-oxide with submodel for riddelliine

;Species : Rat

;Compiled by : Frances Widjaja

;Organisation : Division of Toxicology, Wageningen University and Research, The Netherlands

;================================================================

;Physiological parameters

;================================================================

BW = 0.250 ;body weight rat (kg) (Brown, 1997)

;Tissue fractions (Brown, 1997)

VFc = 0.070 ;fraction of fat tissue

VLc = 0.034 ;fraction of liver

VBc = 0.074 ;fraction of blood

VKc = 0.007 ;fraction of kidney

VRc = 0.041 ;fraction of rapidly perfused tissue

VSc = 0.774 ;fraction of slowly perfused tissue

;total of fractions = 1

;Tissue volumes based on fractions (L or kg)

VF = VFc*BW ;volume of fat tissue

VL = VLc*BW ;volume of liver

VB = VBc*BW ;volume of blood

VK = VKc*BW ;volume of kidney

VR = VRc*BW ;volume of rapidly perfused tissue

VS = VSc*BW ;volume of slowly perfused tissue

;--------------------------------------------------------------------------------------------------------------------

QC = 5.380 ;blood flow rate: cardiac output 15*(BW^0.74) (L/h) (Brown, 1997)

;Fraction of blood flow rate (Brown, 1997)

QFc = 0.070 ;fraction of blood flow to fat tissue

QLc = 0.250 ;fraction of blood flow to liver

QKc = 0.141 ;fraction of blood flow to kidney

QRc = 0.369 ;fraction of blood flow to rapidly perfused tissue (0.76-QLc-QKc)

QSc = 0.170 ;fraction of blood flow to slowly perfused tissue (0.24-QFc)

;total of fractions = 1

;Blood flow rates (L/h)

QF = QFc*QC ;blood flow to fat tissue

QL = QLc*QC ;blood flow to liver

QK = QKc*QC ;blood flow to kidney

QR = QRc*QC ;blood flow to rapidly perfused tissue

QS = QSc*QC ;blood flow to slowly perfused tissue

;================================================================

;Physicochemical Parameters

;================================================================

;Partition coefficients (calculated using QIVIVEtools by WFSR) (Punt, 2020; Berezhkovskiy, 2004)

;Riddelliine N-oxide ; Log Kow -0.4 (xLogP3 3.0)

PF_RIDO_ = 0.14 ; fat/blood partition coefficient RIDO

PL_RIDO_ = 0.76 ; liver/ blood partition coefficient RIDO

PK_RIDO_ = 0.83 ; kidney/blood partition coefficient RIDO

PR_RIDO_ = 0.84 ; rapidly perfused tissues/ blood partition coefficient RIDO

PS_RIDO_ = 0.66 ; slowly perfused tissues/ blood partition coefficient RIDO

;Riddelliine ; Log Kow 0.2 (xLogP3 3.0)

PF_RID_ = 0.15 ; fat/blood partition coefficient RID

PL_RID_ = 0.67 ; liver/ blood partition coefficient RID

PK_RID_ = 0.72 ; kidney/blood partition coefficient RID

PR_RID_ = 0.74 ; rapidly perfused tissues/ blood partition coefficient RID

PS_RID_ = 0.58 ; slowly perfused tissues/ blood partition coefficient of RID

;================================================================

;Kinetic parameters

;================================================================

;Absorption rates (hr-1)

kin1 = 0.46 ; transfer rate of RIDO from lower ileum to cecum (Kimura, 2002)

kin2 = 0.46 ; transfer rate of RID from lower ileum to cecum (Kimura, 2002)

ka1 = 0.23 ; transfer rate of RIDO from small intestine to liver

ka2 = 0.23 ; transfer rate of RIDO from intestinal microbiota compartment to liver (Yang, 2020, Chen, 2018)

kb1 = 0.72 ; transfer rate of RID from small intestine to liver

kb2 = 0.72 ; transfer rate of RID from intestinal microbiota compartment to liver (Chen, 2018)

;To ensure that error1 is 0 and massbalance1 is 1, ensure that kin2 = 0 and kb1=0

;To ensure that error2 is 0 and massbalance2 is 1, ensure that kin1 = 0 and ka1=0

;--------------------------------------------------------------------------------------------------------------------

;Metabolism in intestine

;Large intestine (gut microbiota compartment)

;Scaling factors

FBW = 0.0164 ;fraction of faeces to bodyweight (Hoskins & Zamcheck, 1968)

;Unscaled maximum rate of metabolism (umol hr-1 (g faeces)-1)

VmaxLIM1c = 0.16 ;Vmax for anaerobic RIDO reduction to RID by gut microbiota

;Scaled maximum rate of metabolism (umol hr-1)

VmaxLIM1 = VmaxLIM1c*FBW*BW*1000

;Michaelis Menten constant (umol L-1)

KmLIM1 = 2.63 ;Km for anaerobic RIDO reduction to RID by gut microbiota

;--------------------------------------------------------------------------------------------------------------------

;Metabolism of liver

;Scaling factors

MPL = 35 ;liver microsome protein yield (mg microsome/g liver) (Atio, 1976)

S9L = 143 ;liver S9 fraction (mg S9 protein/g liver) (Punt, 2008)

L = VLc*1000 ;liver (34 g/kg bw)

;Scaled metabolism Vmax/Km of aerobic RIDO reduction to RID (L hr-1)

Lslopec = 0.0005 ;slope of V versus [S] (mL min-1 (mg S9)-1)

Lslope = Lslopec/1000*60*S9L*L*BW ;(umol hr-1)

;Unscaled maximum rate of metabolism (nmol min-1 (mg protein)-1)

VmaxLM2c = 2.093 ;Vmax for RID substrate depletion in incubations with liver microsome (Chen, 2018)

;Scaled maximum rate of metabolism (umol hr-1)

VmaxLM2 = VmaxLM2c/1000*60*MPL*L*BW

;Michaelis Menten constant (umol L-1)

KmLM2 = 75.69 ;Km for RID substrate depletion in liver microsome (Chen, 2018)

;================================================================

;Run settings

;================================================================

;Molecular weight

MWL_RIDO_ = 365.4 ;molecular weight riddelliine N-oxide

MWL_RID_ = 349.38 ;molecular weight riddelliine

;Given oral dose of either riddelliine N-oxide or riddelliine

GDOSE1 = 8.8 ;given RIDO dose (mg/kg bw)

GDOSE2 = 8.4 ;given RID dose (mg/kg bw)

ODOSE1 = GDOSE1*1E-3/MWL_RIDO_*1E6 ;given RIDO dose recalculated (umol/kg bw)

ODOSE2 = GDOSE2*1E-3/MWL_RID_*1E6 ;given RID dose recalculated (umol/kg bw)

F1=0.15 ;assumed bioavailability (either 15% or 100%)

F2=0.15 ;assumed bioavailability (either 15% or 100%)

DOSE1 = ODOSE1*BW*F1 ;total RIDO dose (umol)

DOSE2 = ODOSE2*BW*F2 ;total RID dose (umol)

;Time of exposure

Starttime = 0 ;t-0 (hr)

Stoptime = 24 ;t-final (hr)

;================================================================

;Dynamics

;================================================================

;Small intestine compartment

;ASI_RIDO_ = amount of RIDO remaining in small intestine

ASI_RIDO_'= -kin1*ASI_RIDO_ - ka1*ASI_RIDO_

Init ASI_RIDO_ = DOSE1

;--------------------------------------------------------------------------------------------------------------------

;Intestine compartment

;ALI_RIDO_ = amount of RIDO in large intestine (microbiota compartment) (umol)

ALI_RIDO_' = kin1*ASI_RIDO_ - ALIM1' - ka2*ALI_RIDO_

Init ALI_RIDO_ = 0

CLI_RIDO_ = ALI_RIDO_/(FBW*BW)

;ALIM1 = amount of RIDO reduced to RID in large intestine by gut microbiota (umol)

ALIM1' = VmaxLIM1*CLI_RIDO_/(KmLIM1+CLI_RIDO_)

Init ALIM1 = 0

;--------------------------------------------------------------------------------------------------------------------

;Liver compartment

;AL_RIDO_ = amount of RIDO in liver (umol)

AL_RIDO_' = QL*(CB_RIDO_-CVL_RIDO_) - ALM1' + ka1*ASI_RIDO_ + ka2*ALI_RIDO_

Init AL_RIDO_ = 0

CL_RIDO_ = AL_RIDO_/VL

CVL_RIDO_ = CL_RIDO_/PL_RIDO_

;ALM1 = amount of RIDO reduced to RID in liver in aerobic setting (umol)

ALM1' = Lslope*CVL_RIDO_

Init ALM1 = 0

;--------------------------------------------------------------------------------------------------------------------

;Fat compartment

;AF_RIDO_ = amount of RIDO in fat tissue (umol)

AF_RIDO_' = QF*(CB_RIDO_-CVF_RIDO_)

Init AF_RIDO_ = 0

CF_RIDO_ = AF_RIDO_/VF

CVF_RIDO_ = CF_RIDO_/PF_RIDO_

;--------------------------------------------------------------------------------------------------------------------

;Kidney compartment

;AK_RIDO_ = amount of RIDO in kidney (umol)

AK_RIDO_' = QK*(CB_RIDO_-CVK_RIDO_) - GF_RIDO_'

Init AK_RIDO_ = 0

CK_RIDO_ = AK_RIDO_/VK

CVK_RIDO_ = CK_RIDO_/PK_RIDO_

;GFR = glomerular filtration rate 0.0052*BW*60 (L hr-1) (Walton, 2003)

GFR = 0.08

;GF_RIDO_ = glomerular filtration of RIDO (umol h-1)

GF_RIDO_'=GFR*(CVK_RIDO_*Fub_RIDO_)

Init GF_RIDO_ = 0

;Fub_RIDO_ = fraction unbound of RIDO ([www.qivivetools.wur.nl](http://www.qivivetools.wur.nl) Lobell and Sivarajah, 2003)

Fub_RIDO_ = 0.994

;--------------------------------------------------------------------------------------------------------------------

;Rapidly perfused tissue compartment

;AR_RIDO_ = amount of RIDO in rapidly perfused tissue (umol)

AR_RIDO_' = QR*(CB_RIDO_-CVR_RIDO_)

Init AR_RIDO_ = 0

CR_RIDO_ = AR_RIDO_/VR

CVR_RIDO_ = CR_RIDO_/PR_RIDO_

;--------------------------------------------------------------------------------------------------------------------

;Slowly perfused tissue compartment

;AS_RIDO_ = amount of RIDO in slowly perfused tissue (umol)

AS_RIDO_' = QS*(CB_RIDO_-CVS_RIDO_)

Init AS_RIDO_ = 0

CS_RIDO_ = AS_RIDO_/VS

CVS_RIDO_ = CS_RIDO_/PS_RIDO_

;--------------------------------------------------------------------------------------------------------------------

;Blood compartment

;AB_RIDO_ = amount of RIDO in blood (umol)

AB_RIDO_' = QL*CVL_RIDO_ + QF*CVF_RIDO_ + QK*CVK_RIDO_ + QR*CVR_RIDO_ + QS*CVS_RIDO_ - QC*CB_RIDO_

Init AB_RIDO_ = 0

CB_RIDO_ = AB_RIDO_/VB

;AUC_RIDO_ = area under the curve of RIDO (h umol L-1)

AUC_RIDO_' = CB_RIDO_

Init AUC_RIDO_ = 0

;================================================================

;Sub model compartment of riddelliine

;================================================================

;Small intestine compartment

;ASI_RID_ = amount of RID remaining in small intestine

ASI_RID_'= -kin2*ASI_RID_ - kb1*ASI_RID_

Init ASI_RID_ = DOSE2

;--------------------------------------------------------------------------------------------------------------------

;Intestine compartment

;ALI_RID_ = amount of RID in large intestine (microbiota compartment) (umol)

ALI_RID_' = kin2*ASI_RID_ - kb2*ALI_RID_ + ALIM1'

Init ALI_RID_ = 0

CLI_RID_ = ALI_RID_/(FBW*BW)

;--------------------------------------------------------------------------------------------------------------------

;Liver compartment

;AL_RID_ = amount of RID in liver (umol)

AL_RID_' = QL*(CB_RID_ - CVL_RID_) + kb1*ASI_RID_ + kb2*ALI_RID_ + ALM1' - ALM2'

Init AL_RID_ = 0

CL_RID_ = AL_RID_/VL

CVL_RID_ = CL_RID_ / PL_RID_

;ALM2 = amount of RID metabolized into metabolites in liver (umol)

ALM2' = VmaxLM2*CVL_RID_/(KmLM2+CVL_RID_)

Init ALM2 = 0

;--------------------------------------------------------------------------------------------------------------------

;Kidney compartment

;AK_RID_ = amount of RID in kidney (umol)

AK_RID_' = QK*(CB_RID_-CVK_RID_) - GF_RID_'

Init AK_RID_ = 0

CK_RID_ = AK_RID_/VK

CVK_RID_ = CK_RID_/PK_RID_

;GFR = glomerular filtration rate (L hr-1) (Chang 2015)

GFR = 0.08

;GF_RID_ = glomerular filtration of RID (umol h-1)

GF_RID_'=GFR*(CVK_RID_*Fub_RID_)

Init GF_RID_ = 0

;Fub_RID_ = fraction unbound of RID ([www.qivivetools.wur.nl](http://www.qivivetools.wur.nl) Lobell and Sivarajah, 2003)

Fub_RID_ = 0.71

;--------------------------------------------------------------------------------------------------------------------

;Fat compartment

;AF_RID_ = amount of RID in fat (umol)

AF_RID_' = QF*(CB_RID_-CVF_RID_)

Init AF_RID_ = 0

CF_RID_ = AF_RID_/VF

CVF_RID_ = CF_RID_/PF_RID_

;--------------------------------------------------------------------------------------------------------------------

;Rapidly perfused tissue compartment

;AR_RID_ = amount of RID in rapidly perfused tissue (umol)

AR_RID_' = QR*(CB_RID_-CVR_RID_)

Init AR_RID_ = 0

CR_RID_ = AR_RID_/VR

CVR_RID_ = CR_RID_/PR_RID_

;--------------------------------------------------------------------------------------------------------------------

;Slowly perfused tissue compartment

;AS_RID_ = amount of RID in slowly perfused tissue (umol)

AS_RID_' = QS*(CB_RID_-CVS_RID_)

Init AS_RID_ = 0

CS_RID_ = AS_RID_/VS

CVS_RID_ = CS_RID_/PS_RID_

;--------------------------------------------------------------------------------------------------------------------

;Blood compartment

;AB_RID_ = amount of RID in blood (umol)

AB_RID_' = QL*CVL_RID_ + QK*CVK_RID_ + QF*CVF_RID_ + QR*CVR_RID_ + QS*CVS_RID_ - QC*CB_RID_

Init AB_RID_ = 0

CB_RID_ = AB_RID_/VB

;AUC_RID_ = area under the curve (h umol L-1)

AUC_RID_' = CB_RID_

Init AUC_RID_ = 0

;================================================================

;Mass balance calculations

;================================================================

;RIDO oral exposure

Total1=DOSE1

Calculated1 = ASI_RIDO_ + ALI_RIDO_ + AL_RIDO_ + AF_RIDO_ + AK_RIDO_ + GF_RIDO_ + AR_RIDO_ + AS_RIDO_ + AB_RIDO_ + ALI_RID_ + AL_RID_ + ALM2 + AF_RID_ + AK_RID_ + GF_RID_ + AR_RID_ + AS_RID_ + AB_RID_

ERROR1 = ((Total1-Calculated1)/Total1 + 1E-30)*100

MASSBBAL1 = Total1-Calculated1 +1

;RID oral exposure

Total2=DOSE2

Calculated2 = ASI_RID_ + ALI_RID_ + AL_RID_ + ALM2 + AF_RID_ + AK_RID_ + GF_RID_ + AR_RID_ + AS_RID_ + AB_RID_

ERROR2 = ((Total2-Calculated2)/Total2 + 1E-30)*100

MASSBBAL2 = Total2-Calculated2 +1

;================================================================

;Blood concentrations in ng/mL

;================================================================

CB_RIDO_ngmL = CB_RIDO_*MWL_RIDO_

CB_RID_ngmL = CB_RID_*MWL_RID_

AUC_RIDO_ngmL = AUC_RIDO_*MWL_RIDO_

AUC_RID_ngmL = AUC_RID_*MWL_RID_

;================================================================

**Figure 1S**. Riddelliine N-oxide concentration dependent rate of riddelliine formation in anaerobic incubations with S9 of rat liver. Data are presented as mean ± SD of three independent experiments (n = 3).


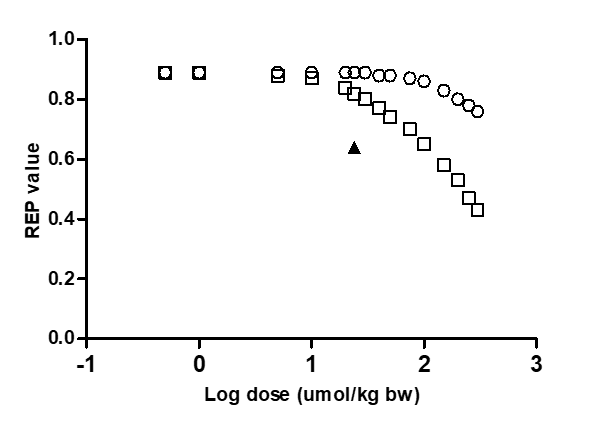


**Figure 2S**. REP value plotted against log equimolar dose (µmol/kg bw) of riddelliine N-oxide and riddelliine as predicted using kinetic parameters from anaerobic liver S9 incubations. White circles represents REP value assuming 15% bioavailability, white squares represents REP value assuming 100% bioavailability, and the black triangle represents the in vivo REP value extracted from Xia et al. (2013) (Xia et al. 2013) (Table 1).
